# Allergenic risk assessment of cowpea and its cross‐reactivity with pea and peanut

**DOI:** 10.1111/pai.13889

**Published:** 2022-12-05

**Authors:** Mouhamed Mounir Chentouh, Françoise Codreanu‐Morel, Aissa Boutebba, Stephanie Kler, Dominique Revets, Annette Kuehn, Markus Ollert, Christiane Hilger

**Affiliations:** ^1^ Department of Biochemistry, Laboratory of Biochemistry and Applied Microbiology University of Badji Mokhtar Annaba Annaba Algeria; ^2^ Department of Infection and Immunity Luxembourg Institute of Health Esch‐Sur‐Alzette Luxembourg; ^3^ Immunology‐Allergology Unit Centre Hospitalier de Luxembourg Luxembourg Luxembourg; ^4^ Department of Dermatology and Allergy Center, Odense Research Center for Anaphylaxis (ORCA) University of Southern Denmark Odense Denmark

**Keywords:** allergenicity, allergens, cowpea, cross‐reactivity, legumes

## Abstract

**Background:**

Novel protein sources can represent a risk for allergic consumers. The aim of this study was to evaluate the allergenicity of cowpea (*Vigna unguiculata*), an increasingly consumed legume and potential new industrial food ingredient which may put legume‐allergic patients at risk.

**Methods:**

Children with allergy to legumes associated to peanut (LP group: *n* = 13) or without peanut allergy (L group: *n* = 14) were recruited and sensitization to several legumes including cowpea was assessed by prick tests and detection of specific IgE (sIgE). Cowpea protein extract was analyzed by SDS‐PAGE and immunoblotting, IgE‐reactive spots were subjected to mass spectrometry. IgE‐cross‐reactivity between cowpea, pea, and peanut was determined using ELISA inhibition assays. Basophil activation tests were performed to evaluate sensitivity and reactivity of patient basophils toward legumes.

**Results:**

Prick tests and sIgE levels to cowpea were positive in 8/14 and 4/13 patients of the L group and in 9/13 and 10/13 patients of the LP group, respectively. Four major IgE‐binding proteins were identified as vicilins and seed albumin. Cowpea extract and its vicilin fraction strongly inhibited IgE‐binding to pea and peanut extract. Peanut, lentil, and pea were the strongest activators of basophils, followed by cowpea, soybean, mung bean, and lupin.

**Conclusion:**

A majority of patients with legume allergy were sensitized to cowpea proteins. Four novel allergens were identified in cowpea, among which storage proteins were playing an important role in IgE‐cross‐reactivity, exposing legume‐allergic patients to the risk of clinical cross‐reactivity to cowpea and thus adding cowpea to the group of nonpriority legumes that are not subjected to allergen labeling such as chickpea, pea, and lentil.

**Key Message**

Allergen labeling regulations require the declaration of peanut and soybean as allergens in food products, but many other legumes are exempt of labeling policies. The introduction of new legumes as protein sources may put legume‐allergic patients at risk due to IgE‐cross‐reactivity. Cowpea, a member of the legume family, showed skin test reactivity, IgE‐binding and basophil activation in patients with legume allergy, pointing to a potential allergic risk of this new protein source. The trend of vegetarian and vegan food requires additional vigilance in patient care and diagnosis.

## 1 INTRODUCTION

In the past few decades, the prevalence of food allergy has been on the rise with up to 10% of the population being affected and a higher prevalence reported in the pediatric population.^1^ In European children and adolescents with food allergy, peanut and nuts are the most prevalent allergen sources.^2^ The frequency of allergy to other legumes, such as soybean, lentil, pea, and chickpea, shows geographic variations based on dietary habits.^3^

Legume seeds and their derived products are increasingly used in food industry due to their nutritional composition and technological properties. They represent an important source of low cost proteins that can be used for the formulation of processed food. Many meals based on legume proteins are present on the market, such as protein bars or meat and dairy product analogs. Pea, lentil, and bean proteins are now frequently used in these products as an alternative for soybean and peanut, which are well known for their allergenicity. However, this alternative is not without danger for the consumers. Indeed, allergies and clinical cross‐reactivities to lentils, pea, chickpea, and beans have already been reported in the past.^3^

Allergen labeling regulations require the declaration of peanut and soybean as allergens in food products. Other legumes, except lupin in Europe and Australia, are exempt of allergen labeling policies. As current and future consumer and food industry trends aim at more sustainable protein sources, more legumes will enter the market, both in terms of quantity and number of varieties. A recent review by Hildebrand et al. analyzed legume allergenicity data available in the literature. The term “non‐priority legumes” was coined for all legumes that are not covered by the current allergen labeling regulations. Significant gaps were identified and further research is urgently needed for nonpriority legumes.^4^

Cowpea, (*Vigna unguiculata*), is a potential new food ingredient entering the European market. It is a significant source of proteins, minerals, and vitamins and is traditionally consumed in Africa, Asia, South America, and also in Southern Europe. Cowpea is now gaining industrial importance in food due to its nutritional and technological characteristics.^5^, ^6^ Cowpea cultivars were evaluated in the EUROLEGUME project for their sustainable farming properties under different agro‐climatic conditions and as ingredient in innovative foods, identifying local genetic resources to be included into breeding programs.^7^

The majority of the identified legume allergens belong to the profilins (e.g., Ara h 5, Gly m 3), the pathogenesis‐related proteins (Ara h 8, Gly m 4), nonspecific lipid transfer proteins (Ara h 9, Len c 3, Pis s 3), 2 S albumins (Ara h 2, Gly m 8), 11 S globulins (Ara h 3, Gly m 6), 7 S globulins (Ara h 1, Gly m 5), and many of them have been well characterized.^8^, ^9^ Seed storage proteins are the most important group of legume food allergens as they can elicit systemic and severe allergic reactions. Vicilins, also named 7 S globulins, belong to the cupin protein superfamily and they are highly abundant seed storage proteins found in legume seeds. Allergic reactions to vicilins were reported for several legumes including pea, peanut, and lentil.^10^, ^11^, ^12^, ^13^ Furthermore, the high amino acid sequence identity between vicilins leads to IgE‐cross‐reactivity between different legumes.^3^ In the Mediterranean area and in India, cross‐reactivity to pea, lentil, and chickpea is common.^14^, ^15^, ^16^, ^17^, ^18^ Patients with an allergy to legumes may have an increased risk when ingesting nonpriority legumes such as cowpea because of potential cross‐reactive molecules common to different legumes. Considering the paucity of our knowledge on nonpriority legumes, this study was initiated to evaluate the sensitization to a panel of different legumes and in particular toward cowpea proteins in a well‐characterized cohort of pediatric patients allergic to legumes, to identify potential new allergens and to evaluate their cross‐reactivity to pea and peanut.

## 2 MATERIAL AND METHODS

### 2.1 Patient recruitment

Legume‐allergic patients were recruited by the Immuno‐Allergology Unit at the Centre Hospitalier de Luxembourg‐KannerKlinik (CHL). All patients presented with a clinical allergy to legumes and had never eaten cowpea before. According to their clinical history, they were divided into two groups depending on whether or not peanut allergic: the first group included patients with allergy to legumes without associated peanut allergy (Legume (L) group). The second group included patients with allergy to peanut and other legumes (Legume/peanut (LP) group), except PA‐23 who had sIgE Ara h 2 at 10 kUA/L, predictive of clinical peanut allergy^19^, ^20^ and PA‐27 who is peanut‐allergic. But both patients were under preventive eviction diet for legumes and clinical symptoms to legumes could not be assessed. Prick‐to‐prick tests were performed with ground dried legume seeds. Total and specific IgE (sIgE) levels were quantified by ImmunoCAP (Thermo Fisher Scientific, Uppsala, Sweden). Skin test and sIgE results were considered as positive when the reaction wheal diameter was ≥3 mm and sIgE levels were ≥0.35 kU_A_/L, respectively. The study was approved by the National Committee for Research Ethics (20180705) and performed according to the standards of the Declaration of Helsinki.

### 2.2 Preparation and analysis of legume protein extracts

Peanut, pea, mung bean, lupin, soybean, lentil, and cowpea seeds were crushed to obtain a finely ground flour. Soluble proteins were extracted using phosphate‐based saline buffer (PBS) (for details, refer to the online repository). An enriched fraction of cowpea vicilin was prepared according to the protocol of de Souza Ferreira et al using successive sodium chloride precipitations.^21^ Protein extracts were separated by 1D and 2D SDS‐PAGE and analyzed by immunoblot. IgE‐binding proteins were excised from the gel and analyzed by MALDI‐TOF MS and MS/MS (online repository).

### 2.3 Quantification of specific IgE by ELISA and ELISA IgE‐inhibition assay

sIgE to cowpea proteins were measured by ELISA as previously described.^22^ For experimental details, refer to the online repository. ELISA inhibition was performed by coating pea or peanut extract and by inhibiting diluted patient sera for 2 h with 200 μg of pea, peanut, or cowpea extract or an enriched vicilin preparation (V) from cowpea before adding to the wells. The inhibition was considered as relevant when IgE‐inhibition was higher than 10%.

### 2.4 Basophil activation test

Basophil activation was performed using the Flow CAST kit (BUHLMANN Laboratories AG) as previously described.^23^ For experimental details, refer to the online repository. Basophil reactivity was determined as the percentage of activated basophils at successive allergen concentrations.

## 3 RESULTS

### 3.1 Legume‐allergic patients, mostly those with peanut allergy, are sensitized to cowpea

Twenty‐seven legume‐allergic patients were recruited in order to evaluate sensitization patterns to several legumes and a potential immunological IgE‐cross‐reactivity between the main allergenic food sources, namely pea and peanut, and cowpea proteins. Mean age was 6 years (range 1–24 years). Patients were divided into two subgroups according to clinical history (Table 1). In group L, 14 patients allergic to legumes but tolerant to peanut were included. Thirteen out of 14 presented with pea or lentil allergy, one patient with mung bean allergy only. In group LP were included 13 patients with an allergy to both legumes and peanut. Many of these allergies to legumes were related to pea and lentil (6 patients), some to soy (2 patients), and 1 to either mung bean, lupin or fenugreek. Clinical symptoms, prick‐to prick‐test results and sIgE levels are listed in Table 1.

**TABLE 1:** Characteristics of legume allergic patients

Patient no	Age (years/sex)	Clinical symptoms	Pea (P)		Peanut (Pn)		Cowpea (C)		Cowpea vicilin	Lentil (L)		Lupin (Lu)		Soybean (S)		CCD
			Skin test (mm)	sIgE (kU_A_/L)	Skin test (mm)	sIgE (kU_A_/L)	Skin test (mm)	sIgE (kU_A_/L)	sIgE (kU_A_/L)	Skin test (mm)	sIgE (kU_A_/L)	Skin test (mm)	sIgE (kU_A_/L)	Skin test (mm)	sIgE (kU_A_/L)	sIgE (kU_A_/L)
Legumes allergic patients without peanut allergy L (*n* = 14)																
PA‐1	6/f	OAS, AP, C (pea, OFC)	8	1.5	7.5	nd	0	<0.35	<0.35	nd	1.3	0	1.2	4	<0.35	nd
PA‐2	5/m	U, V (lentil)	14.5	4.1	2	0.6	7.5	2.6	1.9	13	4.9	15.5	1.5	2	0.9	<0.1
PA‐3	4/f	U, V (lentil)	13	0.4	2	<0.35	3	<0.35	<0.35	4	0.4	3	nd	0	<0.35	<0.1
PA‐4	10/m	OAS (pea, lentil)	10	0.9	0	nd	2	<0.35	<0.35	10	0.6	3.5	<0.35	1	nd	nd
PA‐5	4/f	U, AO (pea)	12	5.2	2	0.9	0	<0.35	<0.35	7	4.2	4	2.6	0	nd	<0.1
PA‐6	1/m	U, Vo, C (pea)	10	3.1	0	0.8	5.5	0.9	0.7	10	2.7	4	1.6	4	0.4	<0.1
PA‐7	5/m	U, AO (pea)	21	12	0	1	0	1.4	2.9	15	13	15	1.5	1	nd	<0.1
PA‐8	3/f	U (pea)	17.5	1.5	1.5	<0.35	4.5	nd	nd	17.5	1.2	8.5	1	2	<0.35	<0.1
PA‐9	6/f	U, AO (pea, lentil)	10	1.9	2	1	3.5	<0.35	<0.35	18	1.3	2.5	0.5	2	0.6	<0.1
PA‐10	9/m	OAS, V (mung bean)	4	0.6	2	1.5	7.5	<0.35	<0.35	8.5	1.2	2	nd	2	2.2	0.7
PA‐11	2/m	U, V, A (pea protein)	7	4.5	0	4.2	5	2.3	1.7	7	5.4	1.5	nd	2.5	2.7	<0.1
PA‐12	7/m	OAS, U (pea, lentil)	11	2	2	3.2	11	<0.35	<0.35	2	1.3	2.5	1.3	2	nd	<0.1
PA‐13	11/m	V (pea)	8	nd	2	<0.35	0	<0.35	<0.35	17	Nd	8	<0.35	4	nd	<0.1
PA‐14	8/m	AO, AP (pea, lentil)	16.5	2.6	0	2.6	0	<0.35	<0.35	10	1.2	0	nd	0	nd	0.7
Prevalence (%)	Female: 36		100	100	7	75	57	31	31	92	100	57	80	21	63	17
Mean	5.8		11.6	3.1	1.6	1.3	3.5	0.6	0.6	10.7	3.0	5.0	1.1	1.9	0.9	0.1
Legumes and peanut allergic patients LP (*n* = 13)																
PA‐15	10/f	OAS, V, AP (peanut) OAS, V (lentil) AP (mung bean)	7	17	10.5	>100	5	63.6	84	4.5	19	1	nd	4	20	nd
PA‐16	6/m	U, V (peanut) U (pea)	7.5	4.2	25	>100	0	0.5	<0.35	13	13	0	nd	2.5	nd	0.6
PA‐17	7/m	AP (peanut) U, AO (pea)	5.5	2.3	17.5	>100	12	0.9	<0.35	12	nd	6.5	1.8	10.5	5.6	0.1
PA‐18	4/m	U (peanut, pea)	21	7.9	13	5.7	9.5	8.6	2.8	18	7.4	8	3.9	9.5	nd	<0.1
PA‐19	11/m	V (peanut) U, A (pea, lentil, soy milk)	7	3.3	7	22	10	<0.35	<0.35	6	nd	8	3.7	7.5	3.2	<0.35
PA‐20	24/m	LAO, V (peanut) LAO (soy bean)	6	3.3	18	51	11	1.9	<0.35	6.5	2.9	5	1	3.5	nd	<0.1
PA‐21	7/f	AP, AO (pea, lentil) AP (peanut)	13	93	13	22	8.5	>100	94.7	22	100	20	35	5	nd	nd
PA‐22	9/f	AP (peanut) AP, U, AO, A (lupin)	10.5	38	17	>100	0	<0.35	<0.35	13	52	13.5	23	6	45	<0.1
PA‐23	4/m	Peanut predictive reactivity	10.5	4.5	10.5	13	4	1.73	0.4	15	4.6	2.5	nd	2	nd	0.5
PA‐24	4/m	U, V, A (peanut) A (lupin)	0	nd	11	91	0	<0.35	<0.35	0	nd	15	2.4	0	nd	<0.1
PA‐25	4/m	OAS, U, AO (peanut) U, R, A (fenugreek)	10	95	17	>100	21	51.9	22.3	13	40	10	14	9.5	40	8.2
PA‐26	4/m	V (peanut), U (soy proteins)	0	nd	21	nd	0	0.6	<0.35	0	nd	14.5	1	4.5	14	<0.1
PA‐27	12/m	OAS, A (peanut)	3	10	37	>100	13.5	10.6	2.2	21	7.5	13	9.2	15.5	5	<0.1
Prevalence %	Female: 23		85	100	100	100	69	77	46	85	100	77	100	77	100	27
Mean	8.2		7.8	25.3	16.7	67.1	7.3	18.5	15.9	11.1	27.4	9	9.5	6.2	19	0.84

*Note*: Skin tests, and sIgE values were considered as positive when the reaction wheal diameters and IgE levels were equal or higher than 3 mm and 0.35 kU_A_/L, respectively. sIgE to cowpea and vicilin were measured by ELISA, all other sIgE by ImmunoCAP. P, pea; Pn, peanut; C, cowpea; L, lentil; Lu, lupin; S, soybean; nd, not done.

Abbreviations: A, Asthma; AO, angioedema; AP, abdominal pain; C, cough; LAO, laryngeal angioedema; OAS, oral allergy syndrome; R, rhinitis; U, urticaria; V, vomiting.

**FIGURE 1:**
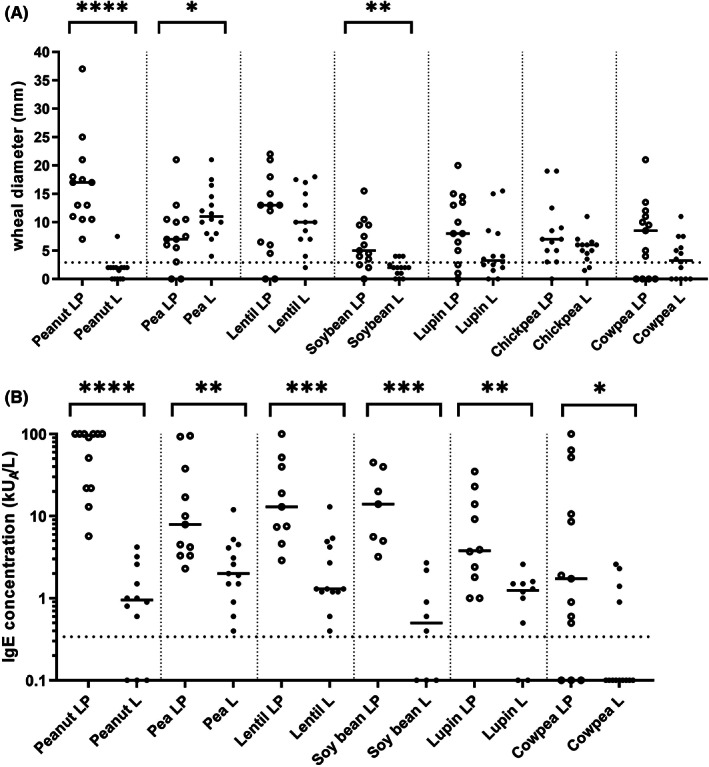
Skin test (A) and sIgE (B) reactivity profiles of patient subgroups. Median values are represented by lines. Dotted line, cut‐off for skin test, and sIgE positivity. Mann–Whitney t‐test was used for the comparison between two subgroups. ****p < .0001; ***p < .001; **p < .01; *p < .05. Only significant *p*‐values are displayed.

Skin test wheal diameters differed between both groups, with a general trend to higher wheal diameters in the LP group, except for pea (Figure 1A). The difference was highly significant for peanut and pea in their respective groups, reflecting the initial clinical group stratification of the participants. The two groups differed also by their sIgE levels. Patients in the LP group had significantly higher levels of sIgE to all legumes, compared to patients of the L group (Figure 1B, Table 1). As a general observation, patients of the LP group have a higher skin reactivity and also higher sIgE levels. Globally, skin tests provided less positive results than sIgE tests, suggesting that prick‐to‐prick tests performed with ground dried seeds may lack relevant legume allergens.

### 3.2 Patients allergic to legumes with or without peanut allergy show IgE‐binding to cowpea proteins

**FIGURE 2:**
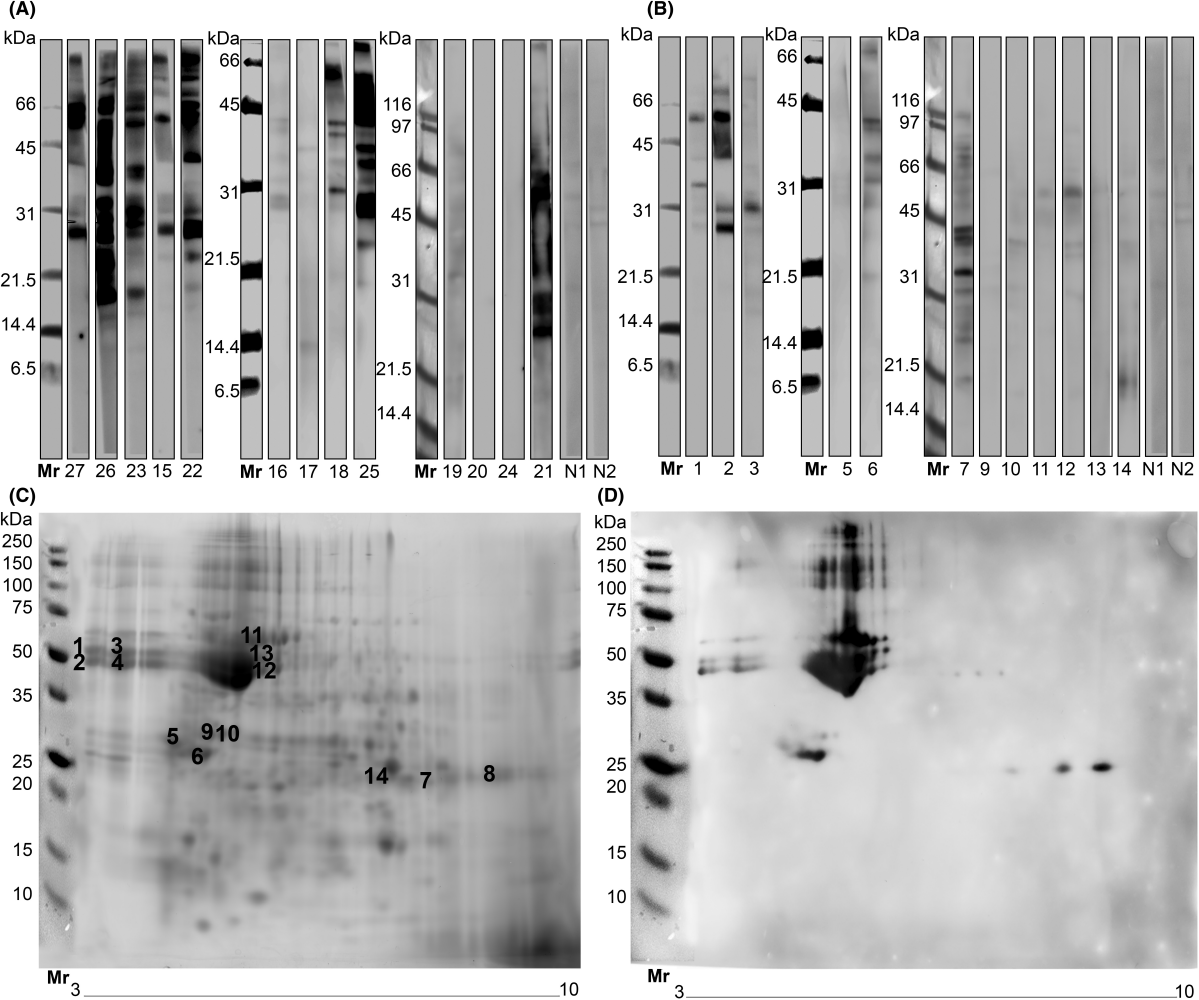
Immunoblot analysis of cowpea protein extracts. First dimension immunoblotting of cowpea crude protein extract under reducing conditions with sera of legume/peanut allergic (LP) patients (A) and legume (L) allergic patients without peanut allergy (B). Blocking buffer (N1) and a serum obtained from a patient not sensitized to legumes (N2) were used as negative controls. 2D‐gel electrophoresis of cowpea crude protein extract stained with SYPRO Ruby protein (C) and second dimension immunoblotting using serum of patient 02 (D). A pH gradient of 3–10 was used for isoelectric focusing. Numbered spots in C correspond to excised protein spots analyzed by MALDI TOF MS/MS.

A potential IgE‐cross‐reactivity toward cowpea proteins was evaluated by IgE‐immunoblot using the sera of legume‐allergic patients (groups L and LP) (Figure 2). Cowpea proteins were recognized by a majority of patient sera of both groups (Figure 2A,B). IgE‐immunoreactive bands were located at a molecular weight ranging from 14 to 80 kDa with major IgE‐binding proteins located at approximately 28 kDa and in the range from 45 to 60 kDa.

### 3.3 7 S vicilins and albumin 2‐like protein are potential IgE‐cross‐reactive allergens in cowpea

IgE‐binding cowpea proteins were identified by 2D‐electrophoresis and immunoblotting using the serum of PA‐02, followed by mass spectrometry (MS) analysis. The choice of this specific patient serum was based on its IgE‐reactivity profile, which is shared among at least 7 patients (Figure 2A,B). In addition, this serum was negative for sIgE to cross‐reactive carbohydrate determinants (CCDs). We were able to identify a total of four IgE‐binding proteins: beta‐conglycinin alpha and beta subunits, and vicilin at a molecular weight ranging from 49 to 60 kDa and albumin‐2 like protein at 29 kDa (Figure 2C,D, Table S1). Several C‐terminal fragments belonging to the beta and alpha subunit of conglycinin were also identified around 30 kDa. These findings suggest that storage proteins consisting mainly of the prolamin and cupin superfamily are mediating the IgE‐cross‐reactivity to cowpea in patients allergic to legumes. The albumin‐2 like protein, vicilin, and the beta‐conglycinin alpha and beta subunits were found to be highly identical to other known legume allergens, sharing percentage identities from 44% to 86%, indicating a potential cross‐reactivity for those allergens with a sequence identity above 50% (Table 2).^24^ An amino acid sequence alignment of cowpea vicilin with homologous allergens from peanut, pea, lentil, soybean, mung bean, and lupin identifies highly conserved regions in all allergen sequences (Figure S1). They correspond to the 2 cupin type‐1 domains in vicilins. Phylogenetic trees have been generated to visually represent the homology between vicilin and 2 S albumin allergens in legumes (Figure S2). Alignments of cowpea vicilin and seed albumin using the 80 amino‐acids sliding window (http://www.allergenonline.org)^25^ yielded even higher identities: 60% with Ara h 1, 69% with Lup an 1, 71% with Pis s 1, 73% with Len c 1, 78% with Gly m 5, and up to 96% for Vig r 4 (data not shown).

**TABLE 2:** Sequence identities of cowpea allergens and related legume allergens and proteins

Cowpea allergen	Similar allergens	Accession number	Species	%Identity
Vicilin (AWS21471, CAP19902.1, A8YQH5)	8 S globulin beta isoform (Vig r 2)	Q198W3	*Vigna radiate*	83.7–85.6
	Alpha subunit of beta conglycinin (Gly m 5)	O22120	*Glycine max*	63.2–64.7
	Convicilin (Pis s 2)	P13915	*Pisum sativum*	53.1–54.0
	Vicilin (Pis s 1)	Q702P1		54.6–55.6
	Gamma‐vicilin subunit (Len c 1)	Q84UI1	*Lenc culiniaris*	57.1–57.6
	Conglutin beta (Lup an 1)	B8Q5G0	*Lupinus angustifolius*	53.7–56.1
	Conglutin beta 1 (Lup a 1)	Q53HY0	*Lupinus albus*	54.4–56.3
	Cupin (Ara h 1)	P43238	*Arachis hypogaea*	48.9–50.6
Beta‐conglycinin beta subunit (XP_027908455, XP_027937581, XP_027937583, XP_027937579)	8 S globulin beta isoform (Vig r 2)	Q198W3	*Vigna radiate*	82.7–86.0
	Alpha subunit of beta conglycinin (Gly m 5)	O22120	*Glycine max*	62.9–64.7
	Convicilin (Pis s 2)	P13915	*Pisum sativum*	50.6–54.0
	Vicilin (Pis s 1)	Q702P1		54.0–56.1
	Gamma‐vicilin subunit (Len c 1)	Q84UI1	*Lenc culiniaris*	55.0–57.9
	Conglutin beta (Lup an 1)	B8Q5G0	*Lupinus angustifolius*	54.1–56.3
	Conglutin beta 1 (Lup a 1)	Q53HY0	*Lupinus albus*	53.8–56.3
	Cupin (Ara h 1)	P43238	*Arachis hypogaea*	49.7–51.6
Beta‐conglycinin alpha subunit (XP_027937578)	8 S globulin beta isoform (Vig r 2)	Q198W3	*Vigna radiate*	68.3
	Alpha subunit of beta conglycinin (Gly m 5)	O22120	*Glycine max*	62.8
	Convicilin (Pis s 2)	P13915	*Pisum sativum*	47.7
	Vicilin (Pis s 1)	Q702P1		55.7
	Gamma‐vicilin sub‐unit (Len c 1)	Q84UI1	*Lenc culiniaris*	57.2
	Conglutin beta (Lup an 1)	B8Q5G0	*Lupinus angustifolius*	46.2
	Conglutin beta 1 (Lup a 1)	Q53HY0	*Lupinus albus*	48.9
	Cupin (Ara h 1)	P43238	*Arachis hypogaea*	44.3
Albumin‐2‐like (XP_027934370)	Seed albumin Vig r 4	Q43680	*Vigna radiate*	87.5

### 3.4 ELISA inhibition studies demonstrate IgE‐cross‐reactivity between pea, peanut, and cowpea

ELISA inhibitions were performed to further evaluate the IgE‐cross‐reactivity between cowpea, pea, and peanut. Soluble proteins were extracted from crude legume flour (Figure S3). In addition, successive sodium chloride precipitations allowed to obtain an enriched vicilin fraction (V) from cowpea, consisting of 2 major bands at 49 and 52 kDa, as well as minor bands at 62, 31, and 24 kDa (Figure S4). The presence of vicilin polypeptides was confirmed by a rabbit anti‐Ara h 1 polyclonal antiserum.

**FIGURE 3:**
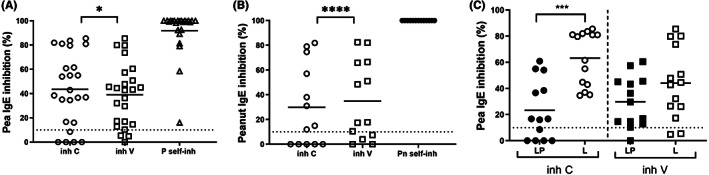
ELISA inhibition of IgE‐binding to pea (A) and peanut proteins (B) using cowpea extract (inh C) and an enriched vicilin fraction (inh V). Comparison of inhibition levels of IgE directed to pea proteins in patient groups LP and L using cowpea and the enriched vicilin fraction (C). Mann Whitney t‐test was used for the comparison between groups (C). Correlations between inhibitions using C and V extracts were calculated by Spearman. Mean values are represented by lines. Dotted line, cutoff for a relevant inhibition. ****p < .0001; ***p < .001; *p < .05.

Inhibition of IgE‐binding to pea and peanut using cowpea extract and enriched vicilins showed that both inhibitors cause a relevant inhibition (>10%) of IgE‐binding in the majority of patients (Figure 3A,B) which demonstrates the presence of IgE‐antibodies cross‐reacting with cowpea proteins. Inhibition of IgE‐binding using either cowpea or vicilin was highly correlated for peanut, moderately for pea (*r* = 0.9282, *p* < .0001 for peanut; *r* = 0.4065, *p* = .0487 for pea), confirming that vicilin was the major allergen responsible for this IgE‐cross‐reactivity (Figure 3A,B). When comparing the inhibition levels of IgE directed to pea proteins in the two patient groups, the inhibition average using cowpea as an inhibitor was found to be significantly higher in the L group (*p* value < .001) (Figure 3C). Vicilin also caused a higher inhibition average in this group, but the difference with the LP group was not significant.

### 3.5 Cowpea allergens are able to activate basophils of patients allergic to legumes and/or peanut

**FIGURE 4:**
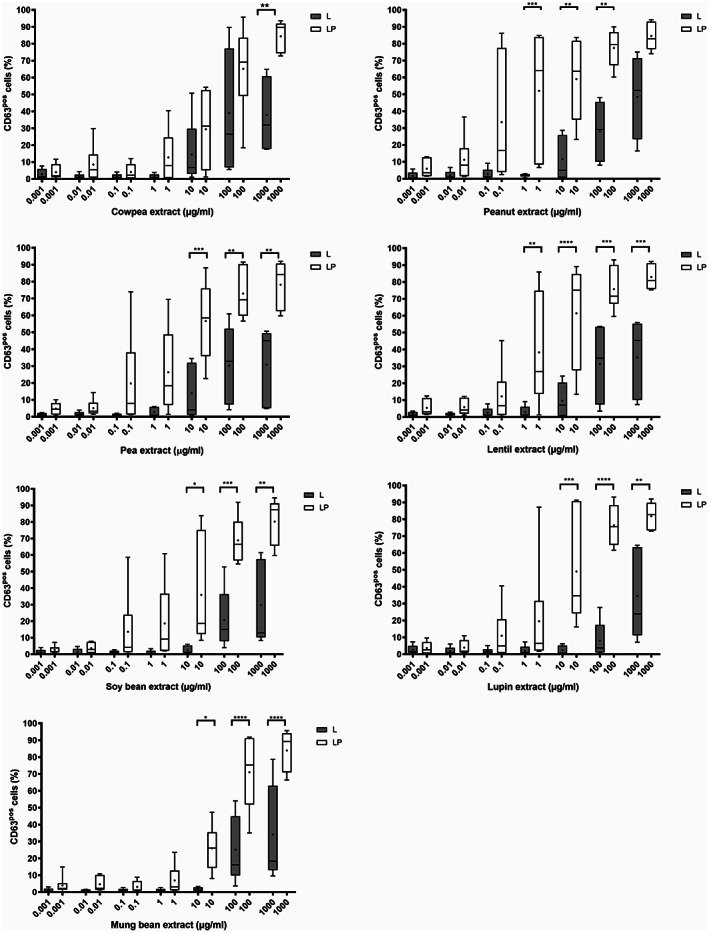
Basophil response to legume crude protein extracts in patients with legume and peanut allergy (LP subgroup) (*n* = 6) and in patients allergic to legumes without peanut allergy (L subgroup) (*n* = 5). Basophils were incubated with increasing concentration of proteins (10^−3^μg/ml to 10^3^μg/ml) from different legumes and relative cell counts of CD63+ basophils were determined by flow cytometry. *p < .05, **p < .01, ***p < .001, and ****p < .0001 for the comparison between groups using Holm‐Sidak's multiple comparisons test (one‐way ANOVA). Median and mean values are represented by lines and crosses, respectively. The cutoff value at 15%, represented by a dotted line, corresponds to the minimal CD63% value for which basophil reactivity is considered positive.

The allergenic potential of cowpea and other legumes was evaluated in basophil activation tests (BAT). All patients demonstrated a significant basophil activation (>15% CD63^+^) to at least one concentration of each extract (Figure 4). Three patients with no allergy to legumes did not show any basophil activation (Figure S5). In the LP group, basophils were activated at lower protein concentrations than in the L group: 0.0227 μg/ml of peanut protein extract was required to induce a significant basophil activation in at least 50% of the LP patients, a concentration of 0.227 μg/ml was needed for pea and lentil while 2.27 μg/ml was required for cowpea, mung bean, soy bean, and lupin, which constitutes a 10 and 100 fold ratio in comparison with peanut. Respective figures in the L group are 227 μg/ml for lupin and 22.7 μg/ml for all other legumes, including peanut. Overall, these findings demonstrate that the basophils of the LP patients are more reactive and more sensitive to all tested legumes. The higher reactivity of this group was statistically significant at high allergen concentrations (Figure 4). Based on the mean basophil response at 2.27 μg/ml of crude protein extract in each group, the following ranking can be established: L patients react most strongly to cowpea, pea, peanut, and lentil, followed by lupin, soybean, and mung bean, (Figure S6). LP patients react most strongly to lentil, pea and peanut. They are less reactive to cowpea, mung bean, soybean, and lupin.

## 4 DISCUSSION

Many members of the seed storage proteins were identified as allergens in legumes. Vicilins and 2 S albumins account for the most important allergens in this family.^3^ In the present study, we identified and characterized new legume allergens from cowpea seeds, and we demonstrated serological and cellular IgE‐cross‐reactivity between cowpea, peanut, and pea.

To our knowledge, no cowpea allergen has been identified so far. Rao et al reported allergic reactions to cowpea that occurred in 6 Indian patients.^26^ The albumin fraction was found to be the main responsible for the allergenicity of cowpea with 2 main immunoreactive protein bands located at 41 and 55 kDa. However, these proteins were not subject to any identification. In the present study, IgE‐immunoblotting analysis of cowpea crude protein extract using legume‐allergic patient sera revealed an immunoreactive pattern with bands ranging from 14 to 80 kDa. Recruited patients were allergic to legumes and either tolerant to peanut (group L) or allergic to peanut (group LP). The majority of the patient sera were reactive to protein bands located at a molecular weight ranging from 44 to 66 kDa. MALDI‐TOF MS and MS/MS analysis of the IgE‐binding proteins identified the beta‐conglycinin alpha subunit (61 kDa), beta conglycinin beta subunit (52 kDa), vicilin (49 kDa) and its proteolytic fragments as new allergens in cowpea. It is known that mature vicilin polypeptides of greater than 50–60 kDa are processed to subunits, this was also reported for pea and lentil vicilins.^12^, ^27^ In addition to the vicilins, another allergen was identified at 25 kDa, the albumin‐2 like protein which is a member of the prolamin superfamily. Homologous allergens to this protein are present in peanut (Ara h 2), soybean (Gly m 8), and mung bean (Vig r 4).

sIgE to vicilin were detected in 71% of the patients with sIgE to cowpea crude protein extract (14 patients). Out of the 17 patients with a positive skin test to cowpea, 11 patients had sIgE to cowpea detected in ELISA and 14 patients were positive in IgE‐immunoblotting. The frequency of IgE sensitivity to cowpea was higher in the LP group and was associated with higher titers of sIgEs to peanut, pea, cowpea, lupin, lentil, and soybean. Full‐length amino acid sequence alignments showed that cowpea allergens are highly identical (up to 86%) to known allergens found in several legumes, including pea, peanut, lentil, soybean, and for which allergenicity is well documented in the literature. The sequence identity described here, in addition to the ELISA inhibition experiments, which show a clear inhibition of the sIgE‐binding to pea and peanut proteins using cowpea or its vicilin fraction, demonstrate that IgE directed to cowpea, pea, and peanut are cross‐reactive. The high sequence identity between vicilins is most likely responsible for the cross‐reactivity observed between legumes and was previously reported in several studies.^11^, ^12^, ^13^, ^27^ Cross‐reactivity to legumes is reported to be clinically significant in countries where a large consumption of legumes is observed.^14^, ^15^, ^16^

In our study, oral food challenges using cowpea seeds were not performed for ethical reasons as these patients usually do not consume cowpea and the direct benefit for subjects was low. This is certainly a weakness of the present report. However, in our study, and for the first time, a large number of legumes were analyzed in the BAT assay. Patients' reactivity and sensitivity toward legumes were evaluated and revealed that basophils of LP patients reacted more strongly to the tested legumes and were more sensitive than basophils of L patients. L patients were also characterized by low sIgE titers and small skin test wheal diameters. Peanut, pea, and lentil were associated to the highest basophil reactivity and sensitivity in the LP group, in agreement with the clinical history. In contrast, for the L group, high concentrations of allergens were needed to significantly activate basophils. This points to the fact that, at least for peanut, a reactivity in this concentration range is not clinically significant, which is in accordance with previous data.^28^ However, relatively high concentrations were also needed for basophil activation using pea and lentil despite the fact that those patients were clinically reactive to pea and/or lentil. The higher basophil reactivity in the LP group could reflect a stronger clinical reaction of these patients, 4/6 reported vomiting upon ingestion of peanut, whereas in the L group, only 2/5 reported vomiting and 3/5 reported angioedema upon ingestion of legumes. As no food challenges were performed, the threshold levels leading to symptoms are not known for our patients, but it has been reported that they also influence basophil reactivity and sensitivity.^29^ Basophil activation profiles have been well established for diagnosis of peanut allergy,^28^, ^30^ but they need further evaluation in the case of legume allergy associated with tolerance to peanut. Cowpea showed a significant basophil reactivity in our patients, sIgE binding to cowpea proteins and positive skin tests in the majority of the patients, pointing to a potential risk of clinical reactivity in polysensitized patients.

A major strength of this study is the analysis of 7 legumes in a large group of patients with a documented clinical history to legumes, and the combination of in vivo, in vitro, in silico and molecular diagnosis tools to establish the allergenicity of these legumes, and of cowpea in particular. The genus Vigna is an important set of legumes consisting of more than 200 species: in Europe, mung beans (*Vigna radiata*), adzuki beans (*Vigna angularis*), and black gram (*Vigna mungo*) are the most commercialized species. Their common names are often inappropriate and may be a source of confusion with soy and beans. Furthermore, cowpea may be misidentified as a bean from Phaseolus genus.

In conclusion, this is the first study to highlight the potential allergic risk of an increasingly commercialized subfamily of legumes, represented here by cowpea. Based on our results, this risk needs to be evaluated by allergists, especially in pea, lentil, and peanut allergic patients. Cowpea could be added to the list of skin prick test to legumes as a representative of the *Vigna* legume subfamily. In the trend of vegan food, additional vigilance needs to be implemented regarding the allergic risk of *Vigna* legumes since they may represent new food allergen sources.

## AUTHOR CONTRIBUTIONS

**Mounir Mouhamed Chentouh:** Formal analysis (lead); funding acquisition (equal); investigation (lead); methodology (equal); validation (equal); writing – original draft (equal); writing – review and editing (equal). **Françoise Codreanu‐Morel:** Conceptualization (equal); investigation (equal); methodology (equal); resources (lead); validation (equal); writing – original draft (equal); writing – review and editing (equal). **Aissa Boutebba:** Funding acquisition (equal); supervision (equal); writing – review and editing (equal). **Stephanie Kler:** Formal analysis (equal); investigation (equal); methodology (equal); validation (equal); writing – original draft (supporting). **Dominique Revets:** Formal analysis (equal); investigation (equal); methodology (equal); validation (equal); visualization (equal); writing – original draft (supporting). **Annette Kuehn:** Validation (equal); writing – original draft (equal); writing – review and editing (equal). **Markus Ollert:** Writing – original draft (equal); writing – review and editing (equal). **Christiane Hilger:** Conceptualization (lead); formal analysis (equal); funding acquisition (equal); project administration (equal); supervision (lead); visualization (equal); writing – original draft (equal); writing – review and editing (equal).

## FUNDING INFORMATION

This work was supported with a short‐term research fellowship from the European Academy of Allergy and Clinical Immunology (EAACI) and with a research grant from the University of Badji Mokhtar Annaba. Supported by the Luxembourg National Research Fund on the PRIDE program grant PRIDE/11012546/NEXTIMMUNE (AK); supported by the Personalized Medicine Consortium grant APSIS, PMC/2017/02 (AK); and by intramural funds of the Ministry of Higher Education and Research (MESR), Luxembourg (AK, MO, CH).

## CONFLICT OF INTEREST

All authors declare that they have no conflicts of interest in regard to the work presented in this study.

## Supporting information


**Appendix S1** Allergenic risk assessment of cowpea and its cross‐reactivity with pea and peanut

Figure S1

Figure S2

Figure S3

Figure S4

Figure S5

Figure S6
