# Supplementary material for: Allergenic risk assessment of cowpea and its cross‐reactivity with pea and peanut
Source: Pediatr Allergy Immunol. 2022 Dec 5;33(12):e13889. doi: 10.1111/pai.13889 (PMC10108199; doi:10.1111/pai.13889)
Supplement: Supplementary file 1 — Appendix S1 Allergenic risk assessment of cowpea and its cross‐reactivity with pea and peanut [file PAI-33-0-s005.docx]

**Supporting information**

**Allergenic risk assessment of a new non-priority legume, cowpea, and its cross-reactivity with pea and peanut**

**Short title: Legume allergic patients are sensitized to cowpea**

Mouhamed Mounir Chentouh^1,2*^, Françoise Codreanu-Morel^3^, Aissa Boutebba^1^, Stephanie Kler^2^, Dominique Revets^2^, Annette Kuehn^2^, Markus Ollert^2,4^, Christiane Hilger^2**^

1. Department of Biochemistry, Laboratory of Biochemistry and Applied Microbiology, University of Badji Mokhtar Annaba, Annaba, Algeria
2. Department of Infection and Immunity, Luxembourg Institute of Health, Esch-Sur-Alzette, Luxembourg
3. Immunology-Allergology Unit, Centre Hospitalier de Luxembourg, Luxembourg, Luxembourg
4. Department of Dermatology and Allergy Center, Odense Research Center for Anaphylaxis (ORCA), University of Southern Denmark, Odense, Denmark

**^*^** MM Chentouh performed the study during a research fellowship at Luxembourg Institute of Health, granted by EAACI

**^**^Corresponding author:**

Christiane Hilger, PhD

Department of Infection and Immunity, Luxembourg Institute of Health (LIH), Esch-sur-Alzette

L-4354, Luxembourg.

Phone : +352 26 970 258; E-mail: [Christiane.Hilger@lih.lu](mailto:Christiane.Hilger@lih.lu)

**Supplementary methods**

**Preparation of legume protein extracts**

Peanut, pea, mung bean, lupin, soybean, lentil and cowpea raw seeds were crushed using a tissue lyser (MM400, Retsch) to obtain a finely ground flour. Soluble proteins were extracted using phosphate-based saline buffer (PBS). 1 g of flour was suspended in 15 ml PBS-buffer and the mixture was continuously stirred at 4°C for 2h. The suspension was then centrifuged at 5000 g for 15 min at 4°C. The supernatant was collected and centrifuged again to eliminate any insoluble material (20,000 g for 15 min at 4°C). Samples were defatted using cold acetone (Aceton:protein extract (4:1)). The pellet, obtained after overnight incubation at -20°C and centrifugation at 20,000 g at 4°C for 15 min, was solubilized in 8M urea containing 2% CHAPS (Sigma). Protein extracts used for BAT were not subject to a defatting step. An enriched fraction of cowpea vicilin was prepared according to the protocol of de Souza Ferreira et al using successive sodium chloride precipitations.^1^ Briefly, cowpea proteins were extracted using a 0.1 M NaCl solution with a pH 7.5 and ratio (1/20). Stirring was applied for 1h at room temperature and was followed by a centrifugation at 10 000g for 40min. A first precipitation of the supernatant was carried by adjusting the pH to 5 and stirring overnight at 4°C, followed by centrifugation (10 000g, 40min). The obtained pellet was extracted again by a NaCl solution (NaCl 0.1M, pH 7, 4°C). The suspension was stirred and centrifuged as above. The supernatant was subjected to a second precipitation and centrifugation in the same conditions as cited above to finally obtain an isolated vicilin fraction in the pellet. A solution made of chapps 2%, urea 8M was used for the solubilization of the pellet.

**1D-Electrophoresis and Immunoblotting**

Protein extracts were analysed by SDS-PAGE using commercial Pre-cast anyKD gels and a PROTEAN TetraCell mini system (Bio-Rad, Temse, Belgium). Extracts (10ug protein) were separated under reducing conditions. Gels were stained using SYPRO Ruby protein gel stain (Invitrogen, ThermoFischer) according to manufacturer’s protocol. The picture was taken using Amersham typhoon gel and blot imaging system (Amersham Typhoon 5, Cytiva). For immunoblotting after electrophoresis, the unstained gel was transferred onto a polyvinylidene difluoride (PVDF) membrane (Millipore, Merk) using BioRad transblot SD-cell equipement (Bio-Rad, Temse, Belgium). PVDF membranes were blocked with 3% bovine serum albumin (BSA) (Sigma) in Tris-buffered saline containing 0.05% Tween 20 (TBST) for 2h at room temperature. After washing the membranes with TBST, patient sera (diluted 1:10 in blocking buffer) were added and then, incubated overnight at 4°C. For detection of human IgE antibodies, membranes were incubated for 2 hours with a HRP-conjugated mouse anti-human IgE monoclonal antibody (Southern Biotech, Antwerpen, Belgium) followed by a washing step and revelation by chemiluminescence using Super signal west femto maximum sensitivity substrate kit (Thermo Fisher scientific, Merelbeke, Belgium).

**2D-Electrophoresis and Immunoblotting**

Cowpea flour was extracted in PBS as described above and defatted using 2D-Clean up Kit (GE Healthcare,Cytiva) according to the manufacturer’s protocol. The protein pellet was dissolved in rehydration buffer (Urea 8M , CHAPS 2%, pH 8.5). Proteins were labelled using Cy5 dye (PRIMETECH, Minsk, Belarus) according to the manufacturer’s protocol. Seven cm nonlinear pH 3-10 IPG strips were rehydrated overnight with 50ug Cy5 labelled protein in rehydration buffer (Urea 8M, CHAPS 0.5%, DTT 10mM, Bio-lytes 0.2%). Proteins were focused using a Protean IEF Cell (Bio-Rad) according to the manufacturer’s instructions. Any kD™ Mini-PROTEAN^®^ TGX™ Precast Protein Gels, were used for the second dimension. Samples were run in triplicate, one gel was stained using SYPRO ruby, and the two other gels were used for IgE immunoblotting as described above.

**MALDI-TOF MS and MS/MS analysis**

After electrophoresis, the 2D gel was stained with Sypro Ruby (Invitrogen) and the proteins of interest were picked under blue light. Proteins were digested using trypsin (Promega, company) (6.25ng/ul in 50mM NH_4_HCO_3_). The digestion occurred at 37°C during 4 h and stopped with 1% trifluoroacetic acid.

Tryptic peptides were directly spotted onto a MALDI target plate (Polished steel 384 target plate, Bruker, Germany), 0.3 μl of the matrix solution (5 mg/mL of alpha-cyano-4-hydroxycinnamic acid, HCCA, Bruker and 1 mg/mL of 2,5-dihydroxybenzoic acid, DHB, Bruker in 50% ACN containing 0.1% TFA) was added to each spot according to the dried droplet method (Bruker manufacturer’s instructions). The tryptic peptides mixtures were analysed using a MALDI TOF/TOF instrument (Ultraflex I, Bruker, Germany). Before each analysis, trypsin digested bovine serum albumin was used for external calibration according to the manufacturer’s instructions.

Proteins were identified by peptide mass fingerprints (PMF). The mass list obtained was used for query (Biotool 3.0) in *Viridipantae* (Green Plants) subset (155528436 sequences) of the Plant_EST database (165622902 sequences; 29491002258 residues), with the following parameters: charge state of +1, mass tolerance 120 ppm, tolerance for 3 missed cleavages, potential cysteine carbamidomethylationoxidation of methionin and MASCOT threshold of 5%.

A Blast (protein-protein) search of the two first mascot results followed by an in silico digestion and a comparison to the spectrum mass list with Biotool Mass Editor (Bruker, Germany) were consolidating the protein identification. The MS/MS database searches were performed with a tolerance of 2 missed cleavages in *Viridiplantae* (Green Plants) subset (155528436 sequences) of the Plant_EST database (165622902 sequences; 29491002258 residues), the mass tolerance for precursor ions was set at 0.5 Da and 0.8 Da for fragment ions and the cut-off score was set to the default MASCOT threshold of 5%.

Protein sequence analysis were performed on protein Basic Local Alignment Search Tool (BLASTp), Constraint-based Multiple Alignment Tool (COBALT) and full-length FASTA search in Allermatch (AllergenDB propeptides removed, version 36.3.8h May, 2020) using the default parameters.

**Quantification of specific IgE by ELISA and IgE-inhibition**

Specific IgE (sIgE) to cowpea proteins were measured by ELISA as previously described.^2^ Briefly, a 96-well microtiter plate (Nunc MaxiSorp, Thermo Fisher Scientific, Waltham, MA) was coated overnight with either crude cowpea extract or an enriched vicilin fraction at 5 μg/mL. Free protein binding sites were blocked using 3% BSA diluted in TBST 0.05%. Patient sera were diluted (1:5) and (1:10) in blocking buffer and then, incubated overnight at 4°C. The sera of 3 healthy adults were used as negative controls. Bound human IgE were detected by incubation with biotin labeled monoclonal anti-human IgE antibodies (Southern Biotechnology) followed by streptavidin-AP (BD Pharmingen, company). Color was developed with para-nitrophenyl phosphate (pNPP) (Sigma) and absorbance was read at 405 nm. A standard curve was created using serial dilutions of serum from a cat-allergic patient with a known level of sIgE against cat serum albumin Fel d 2, determined by ImmunoCAP (ThermoFisher Scientific, Uppsala, Sweden). This serum was added to wells coated with Fel d 2. Cold water fish gelatin (Sigma, Overijse, Belgium) was used as blocking agent (2% diluted in TBST 0.05%). A standard curve was plotted for converting optical density units into specific IgE dilutions and unknown sIgE levels to cowpea allergens were calculated based on this standard curve. Values of less than 0.35 kU_A_/L were considered negative^3^.

ELISA inhibition was performed as explained above except that 100 µl of pea or peanut extract were coated at 5ug/ml to the plate. Inhibitions were made by incubating diluted patient sera for 2h with 200 µg of pea, peanut, or cowpea extract or an enriched vicilin fraction from cowpea before adding to the wells. The inhibition was considered as relevant when IgE inhibition was higher than 10%. An ELISA inhibition experiment to peanut was not performed on pea allergic patient sera due to a relatively low level of peanut sIgE.

**Basophil activation test**

A total of 13 assays could be conducted, based on parental consent for blood sampling and the planning of the medical examination of the children during the course of the study. The blood of three healthy adult volunteers was used as control. Soluble protein extracts were prepared from pea, peanut, cowpea, lentil, soybean and mung bean as described above. They were sequentially diluted and assessed for their ability to induce basophil activation in patients with allergy to legumes. Final protein concentrations in the assay are calculated by adding a dilution factor of 4.4 to the added protein concentration. In vitro basophil activation was performed using the Flow CAST kit (BUHLMANN Laboratories AG, Basel, Switzerland) as previously described.^4^

**References:**

1. De Souza Ferreira E, Capraro J, Sessa F, Demonte A, Consonni A, et al. New molecular features of cowpea bean (Vigna unguiculata, l. Walp) β-vignin. Biosci Biotechnol Biochem. 2018;82:285-291.
2. Hilger C, Dubey VP, Lentz D, Magni C, Revets D, Muller CP,et al. Male-specific submaxillary gland protein, a lipocalin allergen of the golden hamster, differs from the lipocalin allergens of Siberian and Roborovski dwarf hamsters. Int Arch Allergy Immunol. 2015;166:30-40.
3. Swiontek K, Kler S, Lehners C, Ollert M, Hentges F, Hilger C. Component-resolved diagnosis using guinea-pig allergens elucidates allergen sensitization profiles in allergy to furry animals. Clin Exp Allergy. 2021;51:829-835.
4. Mehlich J, Fischer J, Hilger C, Swiontek K, Morisset M, Codreanu-Morel F, et al. The basophil activation test differentiates between patients with alpha-gal syndrome and asymptomatic alpha-gal sensitization. J Allergy Clin Immunol. 2019;143:182-189.

**Table S1 : Cowpea allergens identified by 2D-electrophoresis and IgE-immunoblotting**

| Protein identified | Specie | | Accession number | | Spot number | | Protein part | | Sequence coverage (%) | | Calculated mass | | Observed mass | | pI (calculated) | | pI (observed) | |  |
| --- | --- | --- | --- | --- | --- | --- | --- | --- | --- | --- | --- | --- | --- | --- | --- | --- | --- | --- | --- |
| Beta-conglycinin  beta subunit 1-like | | *Vigna unguiculata* | | XP_027908455 | | 1, 3 | | Full length protein | | \| 62.1, 63.2 \| \| --- \| \|  \| \|  \| \|  \| | | 52.6 | | 53 | | 5.3 | | 3, 3.5 | |
|  |  |  |  | XP_027937579 | | 2 | | Full length protein | | \| 75.1 \| \| --- \| \|  \| \|  \| | | 52.6 | | 49 | | 5.4 | | 3 | |
|  |  |  |  | XP_027937583 | | 4 | | Full length protein | | \| 70.4 \| \| --- \| \|  \| | | 50.8 | | 49 | | 5.3 | | 3.5 | |
|  |  |  |  | XP_027937581 | | 12, 13, 14 | | Full length protein | | 86.7, 72.7, 47.7 | | 51 | | 60, 49, 53 | | 5.3 | | 5.5 | |
|  |  |  |  | XP_027908455 | | 5 | | C-terminal | | 64.9 | | 24.2 | | 29 | | 4.7 | | 4 | |
|  |  |  |  | XP_027937583 | | 6 | | C-terminal | | 48.9 | | 24.1 | | 26 | | 5.1 | | 4.5 | |
| Beta-conglycinin alpha subunit like | *Vigna unguiculata* | | | XP_027937578 | | 10, 11 | | C-terminal | | 39.9, 49.3 | | 25.2 | | 30 | | 5.4 | | 4.8, 5 | |
| Vicilin | | *Vigna unguiculata* | | AWS21471 | | 2, 4, 15 | | Full length protein | | 76.9, 80.6, 67.4 | | 49.8 | | 49 | | 5.2 | | 3, 3.5, 6 | |
|  |  |  |  | CAP19902.1 | | 14 | | Full length protein | | 36.5 | | 49.6 | | 53 | | 5.3 | | 5.5 | |
| Albumin-2 like protein | | *Vigna unguiculata* | | XP_027934370 | | 7 | | Full length protein | | 34.5 | | 25.5 | | 25 | | 6.4 | | 7 | |

**Figure legends:**

**Figure S1**: Amino acid sequence alignment of cowpea vicilin (accession no.A8YQH5) and homologous allergens from mung bean (accession no.Q198W3), soybean (accession no.O22120), pea (accession no.Q702P1), lentil (accession no.Q84UI1), lupin (accession no.B8Q5G0) and peanut (accession no.P43238). The underligned sections correspond to the cupin type-1 conserved domains according to UniProt. Amino acids marked in dark grey mark positions identical in at least 4 sequences.

**Figure S2:** Phylogenetic trees for **(A)** 2S albumin, **(B)** vicilin in cowpea and other commonly consumed legumes. Allergen amino acid sequences were obtained from Pubmed and UniProt using accession codes obtained from the Allergen Nomenclature web site (http://www.allergen.org). The phylogenic trees were generated by introducing the amino acid sequences in FASTA format into MEGA 11 software.

**Figure S3:** SDS-PAGE of legume crude protein extracts (C: cowpea, M: mung bean, L: lentil, Pn: Peanut, Lu: lupin, P: pea, S: soybean). Mr : molecular weight markers are ranging from 6.5 to 200kDa. Protein staining was performed using SYPRO ruby stain.

**Figure S4:** SDS-PAGE of cowpea crude protein extract (C) and the vicilin enriched fraction (V) obtained through successive sodium chloride precipitation. Protein staining was performed using SYPRO ruby (A), vicilin was detected by immunoblot using a rabbit anti-Ara h 1 polyclonal antiserum (B). Molecular weight markers (Mr) are ranging from 6.5 kDa to 200 kDa

**Figure S5:** Basophil response (CD63% pos cells) to legume crude protein extracts in healthy controls (n=3). Each dot is presenting the median value of 3 control samples. The cut-off value at 15% represented by a dotted line corresponds to the minimal CD63% value for which basophil reactivity is considered positive

**Figure S6:** Basophil response (CD63% pos cells) to legume crude protein extracts at 2.27 ug/ml in patients with legume and peanut allergy (LP subgroup) (n = 6) and in patients allergic to legumes without peanut allergy (L subgroup) (n = 4). Median and mean values are represented by lines and crosses respectively. The cut-off value at 15% represented by a dotted line corresponds to the minimal CD63% value for which basophil reactivity is considered positive.
