# Supplementary material for: Allergenic risk assessment of cowpea and its cross‐reactivity with pea and peanut
Source: Pediatr Allergy Immunol. 2022 Dec 5;33(12):e13889. doi: 10.1111/pai.13889 (PMC10108199; doi:10.1111/pai.13889)
Supplement: Supplementary file 2 — Figure S1 [file PAI-33-0-s001.pdf]

```
                            10         20         30         40         50         60         70         80         90        100
                    ....|....| ....|....| ....|....| ....|....| ....|....| ....|....| ....|....| ....|....| ....|....| ....|....|
A8YQH5_VIGUN        ---------- ---------- ---------- ---------- ---------- ---------- ---------- ---------- ---------- ----------
Q198W3_VIGRA        ---------- ---------- ---------- ---------- ---------- ---------- ---------- ---------- ---------- ----------
O22120_SOYBN        ---------- ------VEKE ECEEGEIPRP RPRPQHPERE PQQPGEKEED EDEQPRPIPF PRPQPRQEEE H--EQREEQE WPRKEEKRGE KGSEEEDEDE
Q702P1_PEA          ---------- ---------- ---------- ---------- ---------- ---------- ---------- ---------- ---------- ----------
Q84UI1_LENCU        ---------- ---------- ---------- ---------- ---------- ---------- ---------- ---------- ---------- ----------
B8Q5G0_LUPAN        ---------E KDFTKNPPKE REEEEHEPRQ QPRPRQQEEQ EREHRREEKH DGEPSRGR-- SQSEESQEEE H--ERRREHH REREQEQQPR PQRRQEEEEE
P43238|ALL12_ARAHY  KSSPYQKKTE NPCAQRCLQS CQQEPDDLKQ KACESRCTKL EYDPRCVYDP RGHTGTTNQR SPPGERTRGR QPGDYDDDRR QPRREEGGRW GPAGPRERER

                           110        120        130        140        150        160        170        180        190        200
                    ....|....| ....|....| ....|....| ....|....| ....|....| ....|....| ....|....| ....|....| ....|....| ....|....|
A8YQH5_VIGUN        ---------- ---IVHRGHQ ESQEESEPRG Q--------- ---------- ---------- -----NNPFY FDSDRWFHTL FRNQYGHLRV LQRFDQRSKQ
Q198W3_VIGRA        ---------- ---IVHREHQ ESQEESDSRG Q--------- ---------- ---------- -----NNPFY FNSDRRFHTL FKNQYGHLRV IHRFDQRSKQ
O22120_SOYBN        DEEQDERQFP FPRPPHQKEE RKQEEDEDEE QQR------- ---------- ESEESEDSEL RRHKNKNPFL FGSNR-FETL FKNQYGRIRV LQRFNQRSPQ
Q702P1_PEA          ---------- ---------S RSDQE----- ---------- ---------- ---------- ------NPFI FKSNR-FQTL YENENGHIRL LQKFDKRSKI
Q84UI1_LENCU        ---------- ---------S RSDQE----- ---------- ---------- ---------- ------NPFI FKSNR-FQTI YENENGHIRL LQRFDKRSKI
B8Q5G0_LUPAN        EEEWQPRRQR PQSRREEREE REQEQGSSSG SQRGGGDERR QHRERRVHRE EREQEQDSRS DSRRQRNPYH FSSNR-FQTY YRNRNGQIRV LERFNQRTNR
P43238|ALL12_ARAHY  EEDWRQPRED WRRPSHQQPR KIRPEGREGE QEWG------ ---------- ---TPGSHVR EETSRNNPFY FPSRR-FSTR YGNQNGRIRV LQRFDQRSRQ

                           210        220        230        240        250        260        270        280        290        300
                    ....|....| ....|....| ....|....| ....|....| ....|....| ....|....| ....|....| ....|....| ....|....| ....|....|
A8YQH5_VIGUN        IQNLENYRVV EFQSKPNTLL LPHHADADFL LVVLNGRAIL TLVNPDGRDS YILEQGHAQK TPAGTTFFLV NHDDNENLRI VKLAVPVNNP HRFQDFFLSS
Q198W3_VIGRA        IQNLENYRVV EFKSKPNTLL LPHHADADFL LVVLNGRAIL TLVNPDGRDS YILEQGHAQK IPAGTTFFLV NPNDNDNLRI IKLAIPVNNP HRFQNFFLSS
O22120_SOYBN        LQNLRDYRIL EFNSKPNTLL LPNHADADYL IVILNGTAIL SLVNNDDRDS YRLQSGDALR VPSGTTYYVV NPDNNENLRL ITLAIPVNKP GRFESFFLSS
Q702P1_PEA          FENLQNYRLL EYKSKPHTLF LPQYTDADFI LVVLSGKATL TVLKSNDRNS FNLERGDAIK LPAGTIAYLA NRDDNEDLRV LDLAIPVNKP GQLQSFLLSG
Q84UI1_LENCU        FENLQNYRLL EYKSKPHTIF LPQFTDADFI LVVLSGKAIL TVLNSNDRNS FNLERGDTIK LPAGTIAYLA NRDDNEDLRV LDLAIPVNRP GQLQSFLLSG
B8Q5G0_LUPAN        LENLQNYRII EFQSKPNTLI LPKHSDADFI LVVLNGRATI TIVNPDKRQV YNLEQGDALR LPAGTTSYIL NPDDNQNLRV AKLAIPINNP GKLYDFYPST
P43238|ALL12_ARAHY  FQNLQNHRIV QIEAKPNTLV LPKHADADNI LVIQQGQATV TVANGNNRKS FNLDEGHALR IPSGFISYIL NRHDNQNLRV AKISMPVNTP GQFEDFFPAS

                           310        320        330        340        350        360        370        380        390        400
                    ....|....| ....|....| ....|....| ....|....| ....|....| ....|....| ....|....| ....|....| ....|....| ....|....|
A8YQH5_VIGUN        TEAQQSYLQG FSKNILEASF DSDFKEINRV LFGEEE--QK QQDEES---- ----QQEGVI VQLKREQIRE LMKHAKSTSK KSLSTQ---N EPFNLRSQKP
Q198W3_VIGRA        TEAQQSYLRG FSKNILEASF DSDFKEIDRV LFGEER--QQ QHGEES---- ----QEEGVI VELKREQIRE LIKHAKSSSR KELSSQ---D EPFNLRNSNP
O22120_SOYBN        TEAQQSYLQG FSRNILEASY DTKFEEINKV LFSREE--GQ QQGEQR---- ----LQESVI VEISKEQIRA LSKRAKSSSR KTISSE---D KPFNLRSRDP
Q702P1_PEA          TQNQPSLLSG FSKNILEAAF NTNYEEIEKV LLEQQE--QE PQHRRSLKDR RQEINEENVI VKVSREQIEE LSKNAKSSSK KSVSSE---S GPFNLRSRNP
Q84UI1_LENCU        TQNQPSFLSG FSKNILEAAF NTEYEEIEKV LLEEQE--QK SQHRRSLRDK RQEITNEDVI VKVSREQIEE LSKNAKSSSK KSVSSE---S EPFNLRSRNP
B8Q5G0_LUPAN        TKDQQSYFSG FSKNTLEATF NTRYEEIERV LLGDDE--LQ ENEKQRR-GQ EQSHQDEGVI VRVSKKQIQE LRKHAQSSSG EGKPSE---S GPFNLRSNKP
P43238|ALL12_ARAHY  SRDQSSYLQG FSRNTLEAAF NAEFNEIRRV LLEENAGGEQ EERGQRRWST RSSENNEGVI VKVSKEHVEE LTKHAKSVSK KGSEEEGDIT NPINLREGEP

                           410        420        430        440        450        460        470        480        490        500
                    ....|....| ....|....| ....|....| ....|....| ....|....| ....|....| ....|....| ....|....| ....|....| ....|....|
A8YQH5_VIGUN        IYSNKFGRLH EITPEK-NPQ LRDLDVFLTS VDIKEGGLLM PNYNSKAIVI LVVNKGEANI ELVGQR-EQQ QQQQ--EESW E--------- -VQRYRAEVS
Q198W3_VIGRA        IYSNKFGRWY EITPEK-NPQ LKDLDVFISS VDMKEGGLLL PHYNSKAIVI LVINEGEAKI ELVGPS-DQQ QQ----DESL E--------- -VQRYRAELS
O22120_SOYBN        IYSNKLGKFF EITPEK-NPQ LRDLDIFLSI VDMNEGALLL PHFNSKAIVI LVINEGDANI ELVGLK-EQQ QEQQQEEQPL E--------- -VRKYRAELS
Q702P1_PEA          IYSNKFGKFF EITPEK-NQQ LQDLDIFVNS VDIKEGSLLL PNYNSRAIVI VTVTEGKGDF ELVGQR-NEN Q---GKENDK EEEQEEETSK QVQLYRAKLS
Q84UI1_LENCU        IYSNKFGKFF EITPEK-NPQ LQDLDIFVNS VEIKEGSLLL PNYNSRAIVI VTVNEGKGDF ELVGQR-NEN QQEQREENDE EEGQEEETTK QVQRYRARLS
B8Q5G0_LUPAN        IYSNKFGNFY EITPDI-NPQ FQDLNISLTF TEINEGALLL PHYNSKAIFI VVVDEGEGNY ELVGIR-DQQ RQQDEQEEEY EQGEEE---- -VRRYSDKLS
P43238|ALL12_ARAHY  DLSNNFGKLF EVKPDKKNPQ LQDLDMMLTC VEIKEGALML PHFNSKAMVI VVVNKGTGNL ELVAVRKEQQ QRGRREEEED EDEEEEGSNR EVRRYTARLK

                           510        520        530        540        550        560        570        580        590        600
                    ....|....| ....|....| ....|....| ....|....| ....|....| ....|....| ....|....| ....|....| ....|....| ....|....|
A8YQH5_VIGUN        DDDVFVIPAS YPVAITATSN LNFIAFGINA ENNQRNFLAG EEDNVMSEIP TEVLDVTFPA SGEKVEKLIN KQSDSHFTDH SSKREER--- ----------
Q198W3_VIGRA        EDDVFVIPAA YPVAINATSN LNFFAFGINA ENNQRNFLAG EKDNVMSEIP TEVLDVSFPA SGNKVEKLIK KQSESHFVDA QPEQQQR--- ----------
O22120_SOYBN        EQDIFVIPAG YPVVVNATSN LNFFAIGINA ENNQRNFLAG SQDNVISQIP SQVQELAFPG SAQAVEKLLK NQRESYFVDA QPKKKE---- ----------
Q702P1_PEA          PGDVFVIPAG HPVAINASSD LNLIGFGINA ENNERNFLAG EEDNVISQVE RPVKELAFPG SSHEVDRLLK NQKQSYFANA QPLQRE---- ----------
Q84UI1_LENCU        PGDVLVIPAG HPVAINASSD LNLIGFGINA KNNQRNFLAG EEDNVISQIQ RPVKELAFPG SSREVDRLLT NQKQSHFANA QPLQIE---- ----------
B8Q5G0_LUPAN        KGDVFIIPAG HPLSINASSN LRLLGFGINA NENQRNFLAG SEDNVIKQLD REVKELTFPG SIEDVERLIK NQQQSYFANA QPQQQQR--- ----------
P43238|ALL12_ARAHY  EGDVFIMPAA HPVAINASSE LHLLGFGINA ENNHRIFLAG DKDNVIDQIE KQAKDLAFPG SGEQVEKLIK NQKESHFVSA RPQSQSQSPS SPEKESPEKE

                           610        620
                    ....|....| ....|....| .
A8YQH5_VIGUN        --V------- ---------- -
Q198W3_VIGRA        --EEGHKGRK GSLSSILGSL Y
O22120_SOYBN        ---EGNKGRK GPLSSILRAF Y
Q702P1_PEA          ---------- ---------- -
Q84UI1_LENCU        ---------- ---------- -
B8Q5G0_LUPAN        -EKEGRRGRR GPISSILNAL Y
P43238|ALL12_ARAHY  DQEEENQGGK GPLLSILKAF N
```

Figure S1 : Amino acid sequence alignment of cowpea vicilin (accession no.A8YQH5) and homologous allergens from mung bean (accession no.Q198W3), soybean (accession no.O22120), pea (accession no.Q702P1), lentil (accession no.Q84UI1), lupin (accession no.B8Q5G0) and peanut (accession no.P43238). The underligned sections correspond to the cupin type-1 conserved domains according to UniProt (https://www.uniprot.org/). Amino acids marked in dark grey mark positions identical in at least 4 sequences.
