# Supplementary figures and images for: Allergenic risk assessment of cowpea and its cross‐reactivity with pea and peanut
Source: Pediatr Allergy Immunol. 2022 Dec 5;33(12):e13889. doi: 10.1111/pai.13889 (PMC10108199; doi:10.1111/pai.13889)

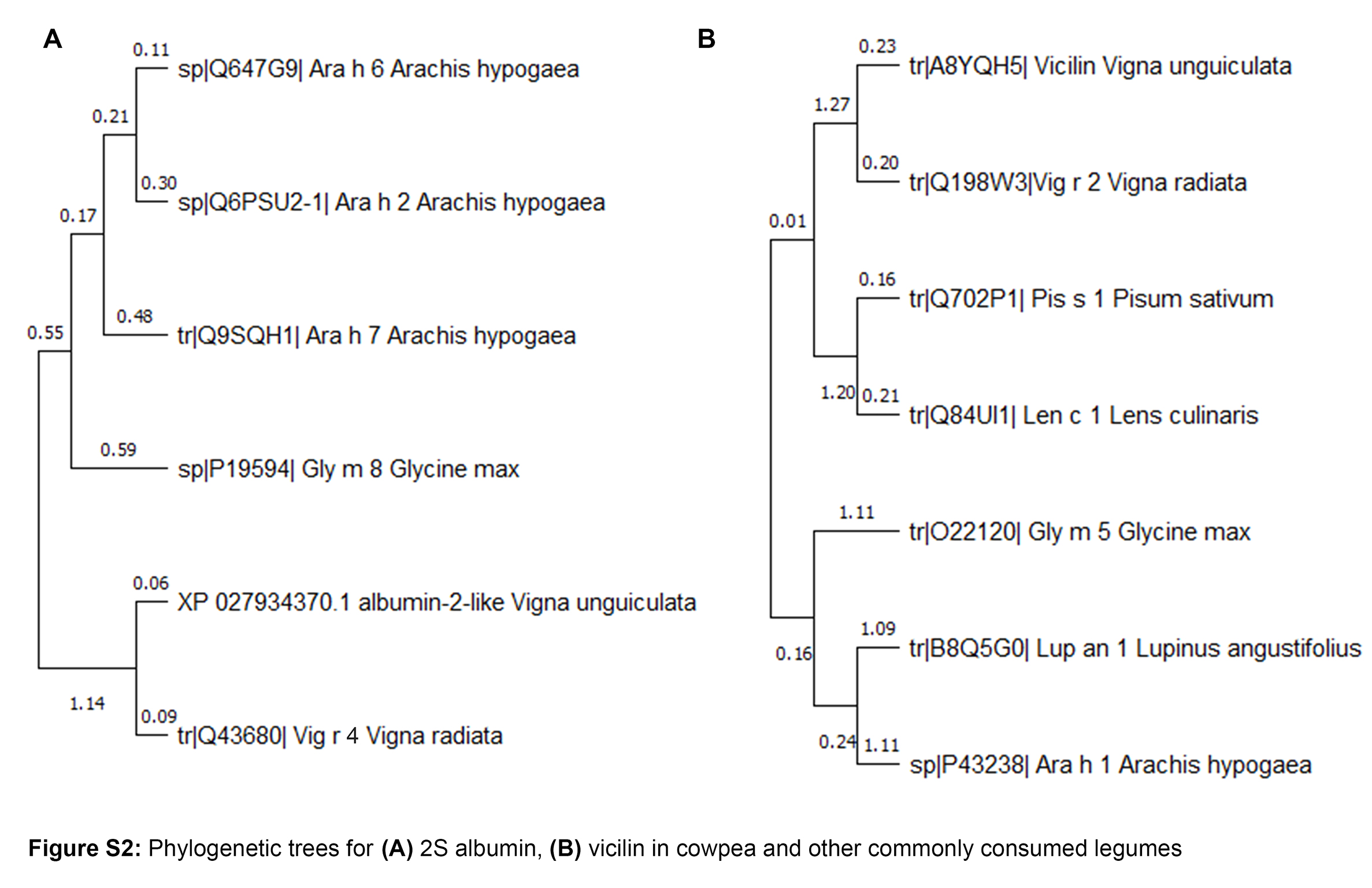

Supplement: Supplementary file 3 — Figure S2 [file PAI-33-0-s006.jpg]

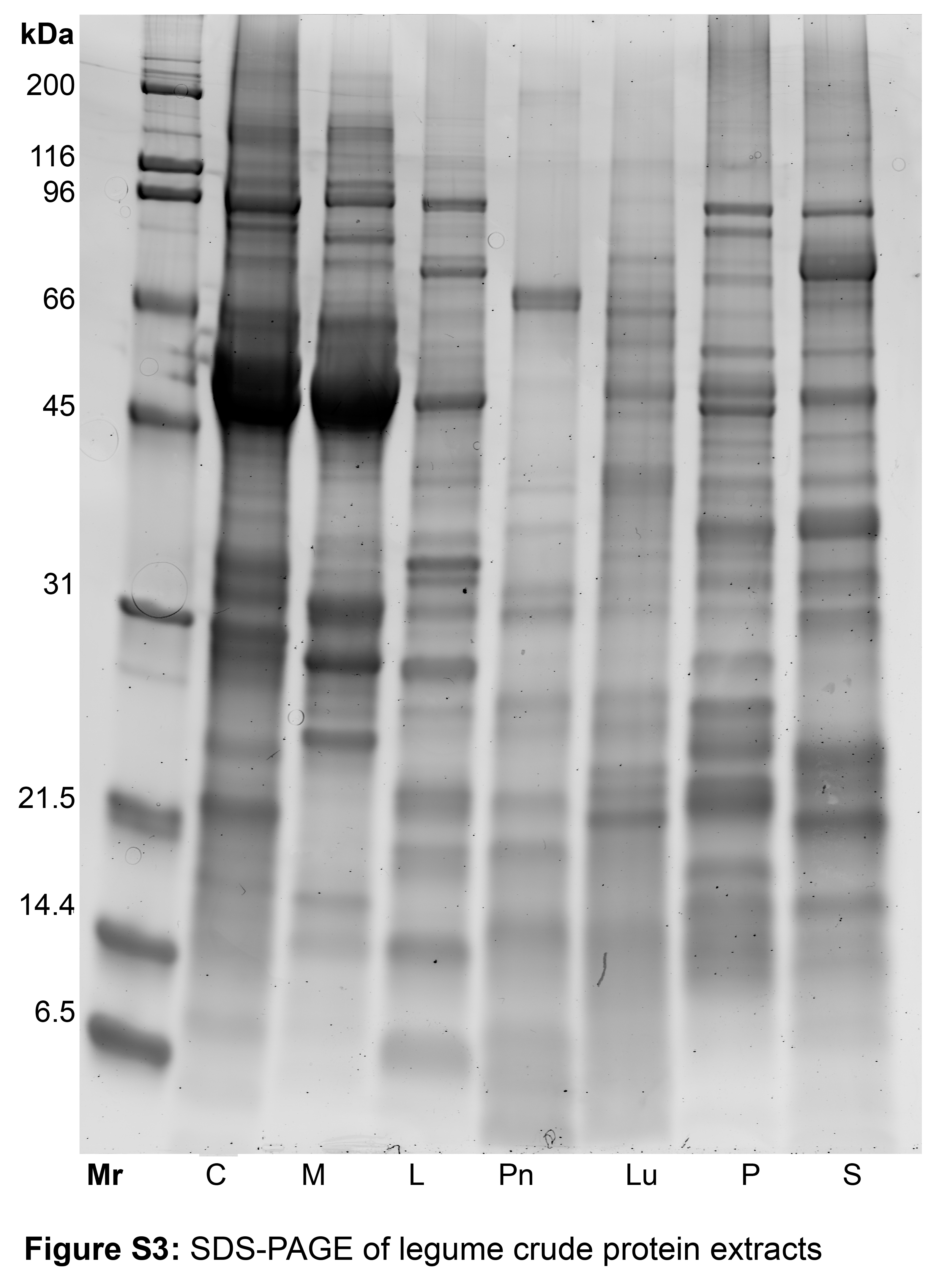

Supplement: Supplementary file 4 — Figure S3 [file PAI-33-0-s003.jpg]

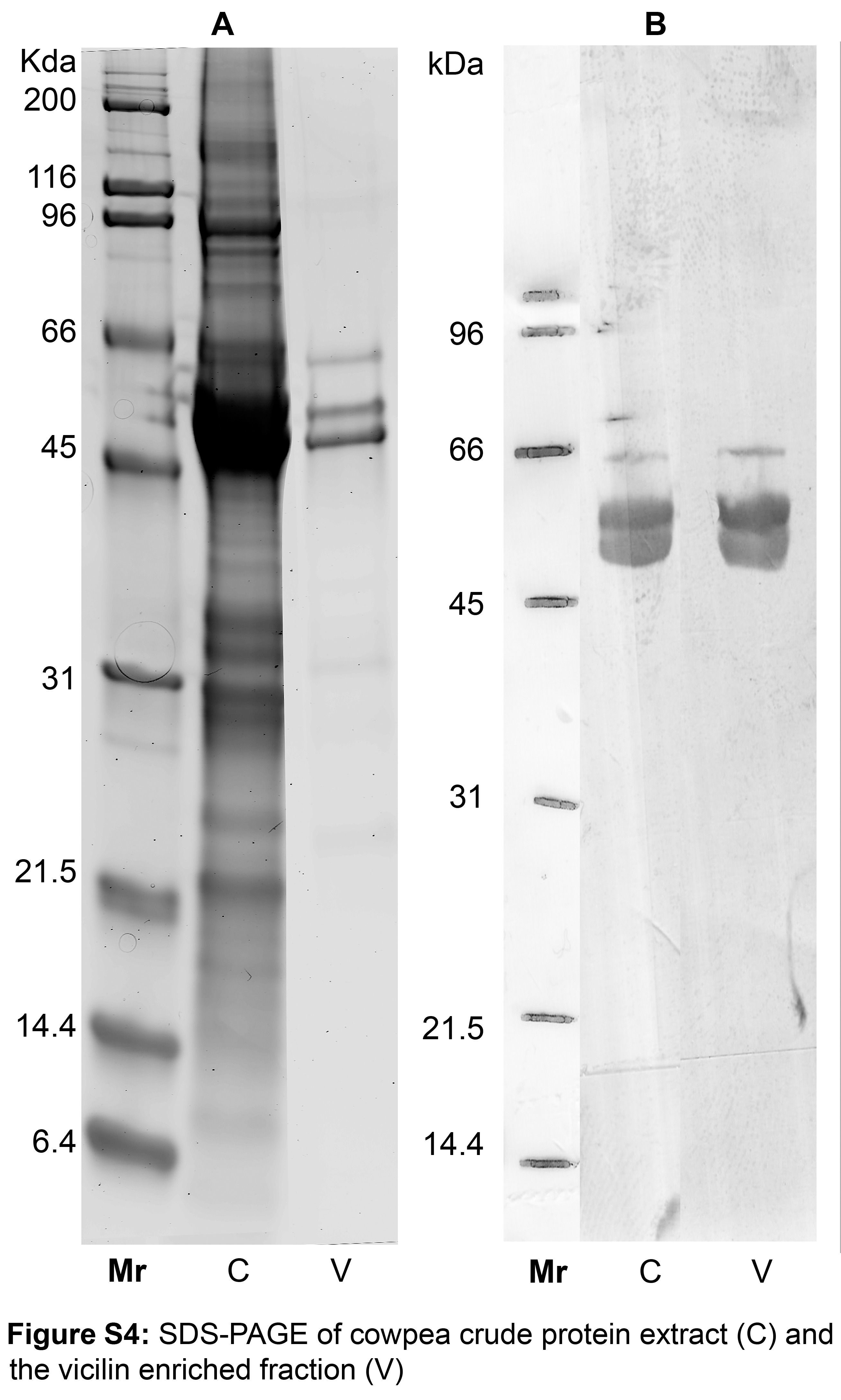

Supplement: Supplementary file 5 — Figure S4 [file PAI-33-0-s004.jpg]

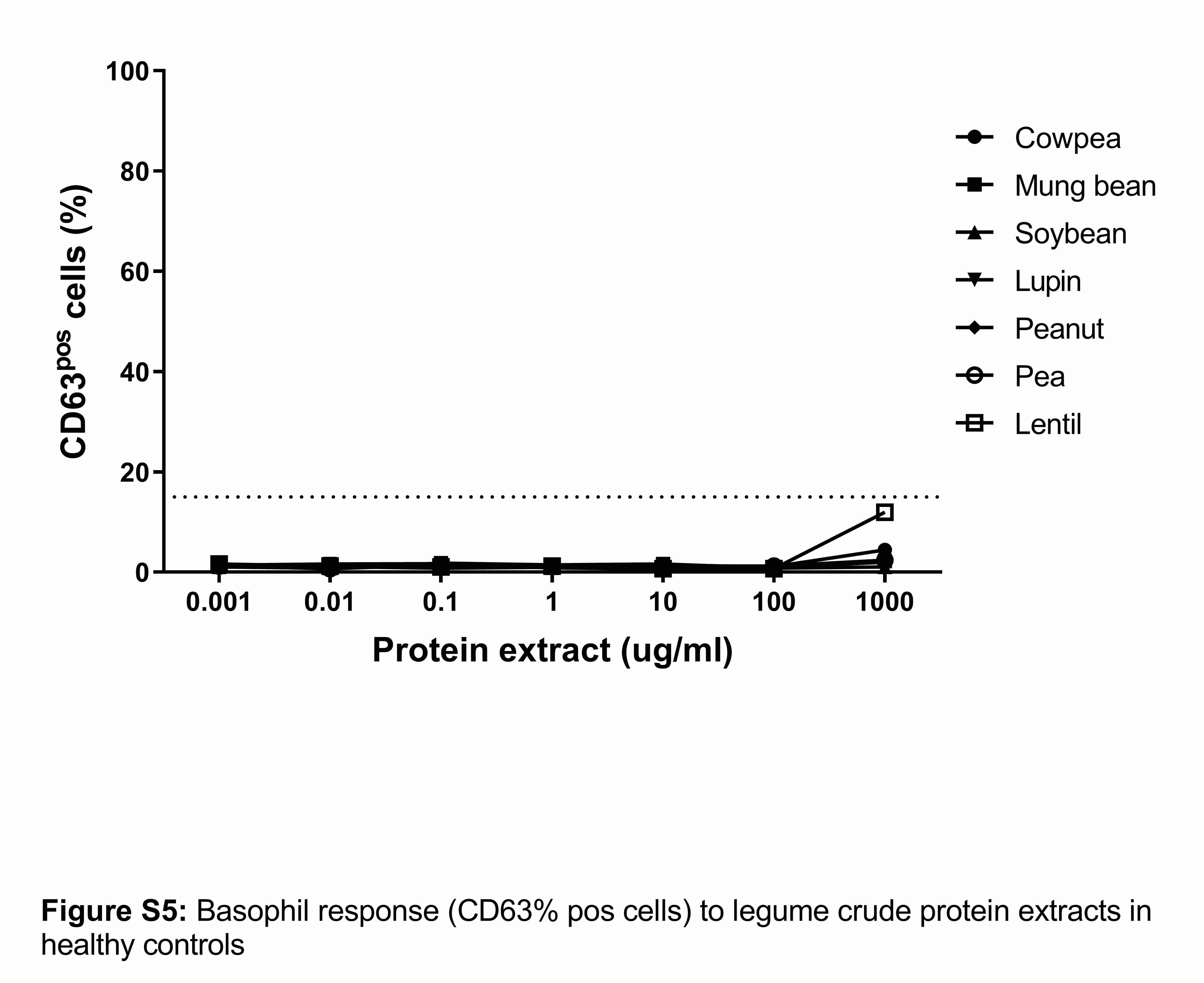

Supplement: Supplementary file 6 — Figure S5 [file PAI-33-0-s007.jpg]

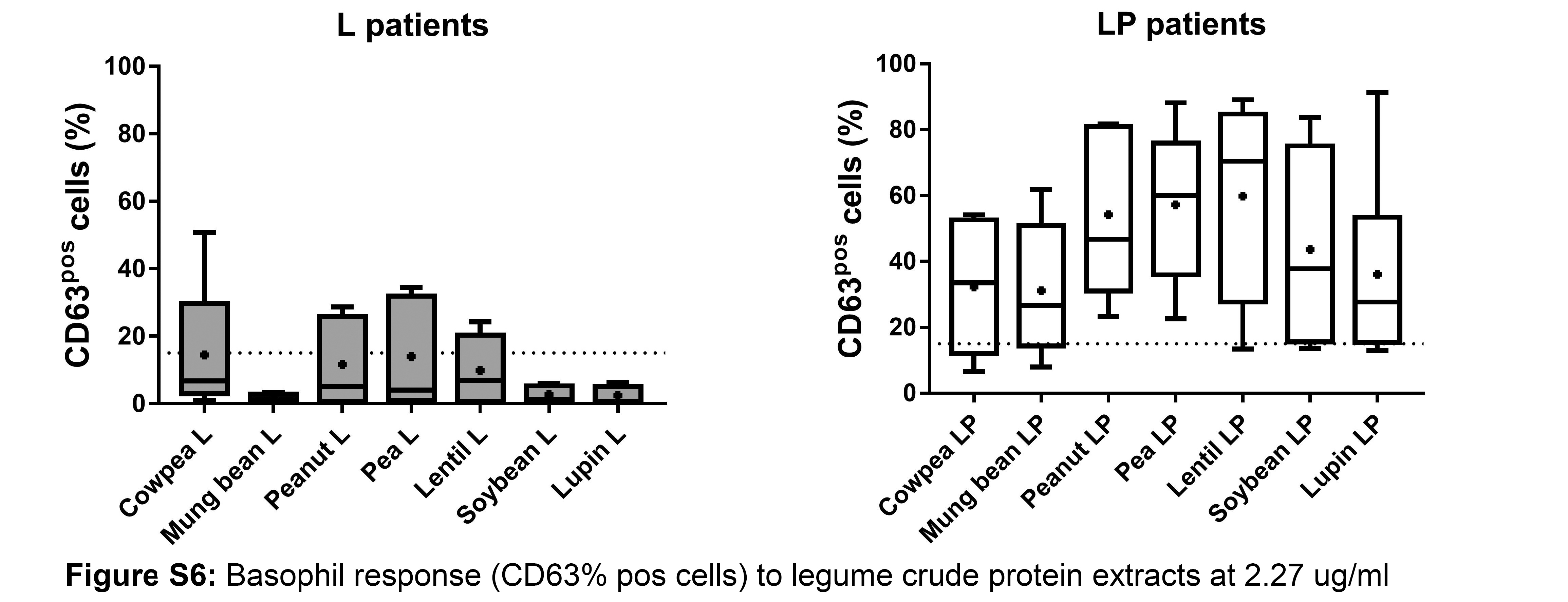

Supplement: Supplementary file 7 — Figure S6 [file PAI-33-0-s002.jpg]
